# Measurement of focal light spot at single-photon level with silicon photomultipliers

**DOI:** 10.1038/s41598-022-17759-y

**Published:** 2022-09-05

**Authors:** Yaxian Yang, Guoqing Zhang, Chen Zhang, Xinyue Cao, Lina Liu, Lianbi Li, Xiaoxiang Han

**Affiliations:** 1 School of Science, Xi’an Polytechnic University, Xi’an, 710048 China; 2 School of Science, Xi’an University of Technology, Xi’an, 710048 China

**Keywords:** Optics and photonics, Physics

## Abstract

Focal spot (light spot) at single-photon level have important applications in many fields. This report demonstrates a method for measuring focal spot size at the single-photon level indirectly. This method utilizes Silicon Photomultiplier (SiPM) as the single-photon sensitive detectors, combined with a nano-positioning stage. The approach involves one- or two-dimensional space scanning and a deconvolution operation, which enable evaluations of the size and spatial distribution of the focal spot formed by a single-photon-level pulsed laser. The results indicate that the average full width at half maximum of the focal spot is about 0.657 μm, which is close to the nominal resolution of the objective lens of the microscope (i.e. 0.42 μm). The proposed method has two key advantages: (1) it can measure focal spot at the single-photon level, and (2) the focal spot can easily be aligned with the detector because the array area of the Geiger mode avalanche photodiode (Gm-APD) cells in SiPM is usually on the order of square millimeter, and there is no need to put an optical slit, knife edge, or pinhole in front of the detector. The method described herein is applicable in weak focal spot detection related fields.

## Introduction

Light spot focusing technology has important applications in laser autocollimation and measurements^1^, two-dimensional exfoliated materials^2^, optical transmission^3^, and biological microfluidic tube preparation^4^. These applications require the light beam to be focused on a small focal spot. Small focal spot at single-photon level, have important applications in time-dependent fluorescence lifetime spectroscopy^5^, confocal laser scanning microscopy (CLSM)^6,7^ and single-photon-emission computed tomography (SPECT)^8^. The conventional methods of measuring light focal spot include plate/plane detector measurements^9–11^, (slit, aperture or knife edge) scanning methods^12,13^, and charge coupling device (CCD) imaging^14–16^. The intensity spatial distribution and size of a millimeter-level focal spot is usually easy to be obtained by these methods directly. Jain et al.^10^ used a digital 194 μm pixel flat panel detector (FPD) combined with a micro pinhole (10 μm) to measure the focal spot, and they applied a deconvolution method to reduce the fuzzy effect caused by the detector during the measurement process; the size of the measured focal spot was about 0.6 mm. However, millimeter-level focal spot cannot meet the spatial accuracy requirements of the above mentioned applications^4^. To obtain smaller light focal spot, Liu et al.^15^ used a CCD to calculate the centroid position of the focal spot based on the two-dimensional (2D) gray values of the small focal spot. This approach allowed the researchers to measure the size of the light focal spot with a diameter on the order of dozens of microns. Tiwari et al.^16^ situated the CCD photosensitive surface perpendicular to the beam axis, recorded the 2D beam intensity distribution on the scanning plane via CCD imaging, and measured a focal spot of 4 μm, which was smaller than the pixel size of the CCD. Nevertheless, the CCD used in this method cannot be applied in single-photon detection, which means that it cannot be used to measure a light focal spot at single-photon level. What's more, to date, measurements of the single photons level have rarely been achieved. Liu ^17^ used a high-speed rotating hollow probe with a small hole to perform arc scanning in the light field and employed a photomultiplier tube (PMT) as the optical signal detector to measure a Bessel beam with a minimum size of 6 μm. With this method, a non-diffractive light focal spot with a small energy density and unknown energy distribution could be measured without focusing, thanks to the high responsiveness, low noise, fast response time, and high quantum efficiency of the PMT. However, the scanning structure was complex, the photon number resolution capability was poor, and the integration level of the system was limited because of the relatively large volume of PMT.

To relieve the aforementioned problems, this report proposes a method for measuring tiny focal spot at the single-photon level, by using Silicon Photomultiplier (SiPM) which has recently been applied in many fields and can be served as a substitute for traditional PMT^18–20^. The SiPM is composed by hundreds to thousands of Single Photon Avalanche Diodes (SPADs), also called Geiger mode APDs (Gm-APDs), each Gm-APD cells is connected in parallel to a common load by a serial resistor with a resistance of about hundreds of kilohm. In this proposed method, the one-dimensional (1D) and 2D spatial distributions of the relative photoresponse of SiPM to the light focal spot were firstly obtained by scanning a nano-positioning stage. Then, the 1D and 2D spatial distributions of the relative intensity of the focal spot under test were determined by deconvolution operation^21^. No slit, knife edge, or pinhole was required in the proposed system.

## Experimental setup and principle

**Figure 1:**
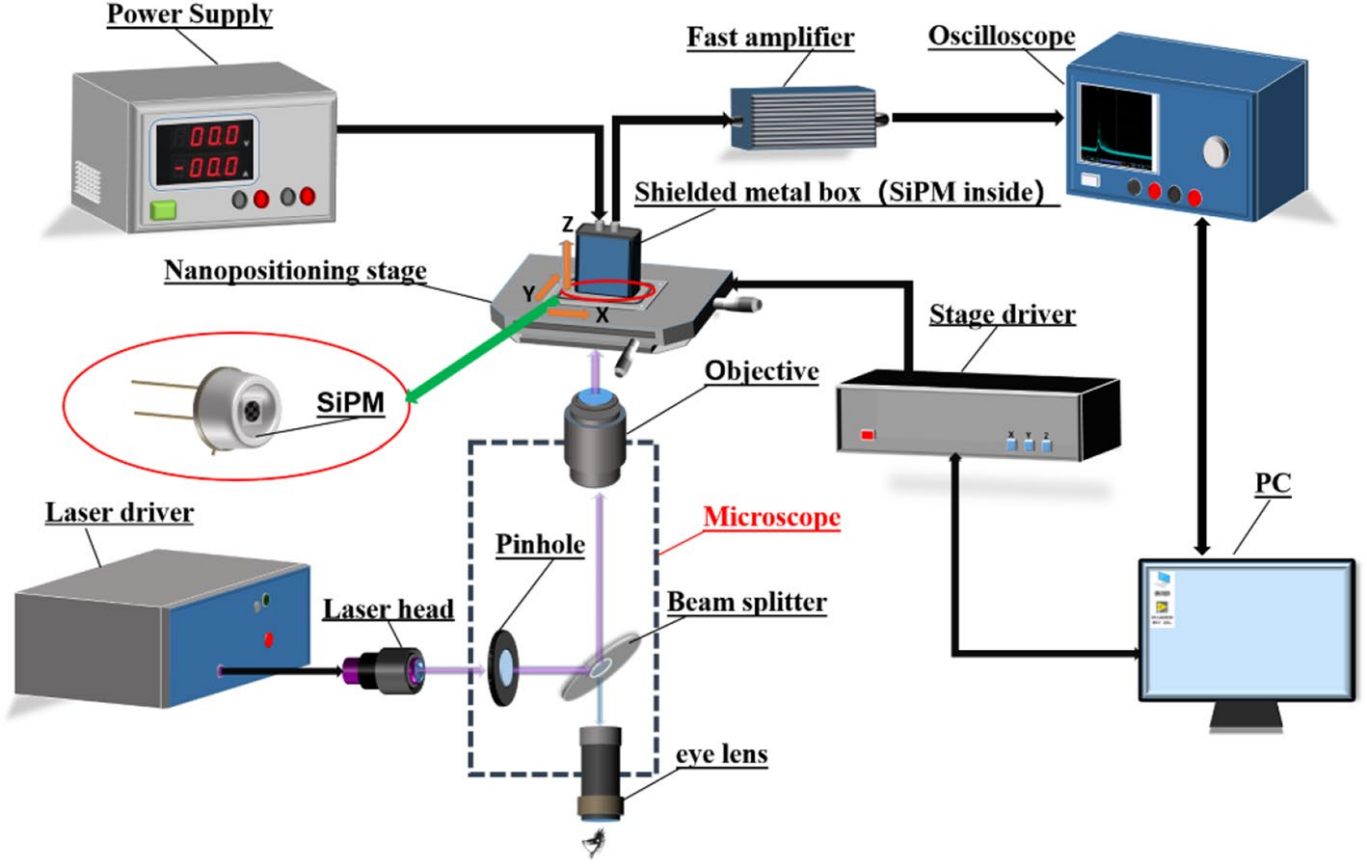
Schematic diagram of the experimental setup. *SiPM* silicon photomultiplier, *PC* personal computer; the dotted box represents the microscope.

The experimental setup used to measure the focal spot is shown in Fig. 1. The SiPM (The model and necessary parameters were shown in Table 1) was placed in a shielded metal box with an optical aperture. The photosensitive side of the SiPM was fixed facing downward on the nano-positioning stage (closed-loop displacement accuracy = 2 nm; displacement range = 200 μm; PI nanoXYZ®, Germany), which facilitated alignment with the laser spot. The nano-positioning stage was controlled by the stage driver, which could freely move towards the *x-*, *y-*, and *z-*directions along with the SiPM. The bias voltage for SiPM from a power supply is several volts higher than the SiPM’s breakdown voltage (the over-voltage was 3 V in this work). The output avalanche pulse signal from SiPM was first amplified by a fast amplifier (homemade, bandwidth = 10 kHz–1 GHz, Gain = 50), and then sent into a high-speed digital storage oscilloscope (Tektronix DPO4102B-L digital phosphor oscilloscope, USA; sample rate = 5 GHz; bandwidth = 1 GHz) to record the pulse waveform and pulse counting rate (*PCR*). The threshold of the oscilloscope was set to 0.5 p.e. (where p.e. = photon equivalent) to eliminate the electronic noise from the avalanche pulses, and the hold off time of the oscilloscope was set larger than the recovery time of the Gm-APD cell of SiPMs to eliminate most of the after-pulse and delay crosstalk event. The laser beam generated by a pico-second pulsed laser (PDL-800D; central wavelength = 375 nm; full width at half maximum (FWHM) in the time domain = 44 ps; repetition frequency = 31.125 kHz to 80 MHz; maximum average optical power = 0.7 mW, PicoQuant GmbH Inc., Germany) was firstly passed through a 100 μm pinhole to limit the diameter and intensity of the light beam and then reflected by a beam- splitter of the microscope (Olympus X-73, Japan).Then, the laser beam was focused through the objective lens (Model: LMPLFLN, Japan; 100×; resolution = 0.42 μm, Olympus Corp., Japan), thus forming a tiny light spot on the surface of the SiPM. The intensity of the pulsed laser was adjusted to attenuate the number of photons reaching the photosensitive surface of the SiPM to the single-photon level for each light pulse, which means that there were only a few photons in each laser pulse on average. The distance between the SiPM and the objective lens (i.e. the z position of the nano-positioning stage) should be optimized after changing SiPM. The SiPM was scanned row-by-row by controlling the nano-positioning stage, and the SiPM output pulse counting rate (*PCR*) at each position in the *x*- and *y*-directions was recorded to obtain the data matrix of the *PCR*. The 1D and 2D *PCR* distribution maps could then be constructed using any common mathematical software.

**Table 1:** The parameters of the SiPMs used in this report^22–25^.

Model of SiPM	Gm-APD cell pitch (μm)	Total area of SiPM(mm^2^)	*V*_bd_ (Breakdown voltage) (V)
FBK SiPM (NUV-HD-LF)	35	3 × 3	32.50
NDL SiPM(EQR1011-1010C-T)	10	1 × 1	26.50
Hamamatsu(S12571-010C)	10	1 × 1	66.00

**Figure 2:**
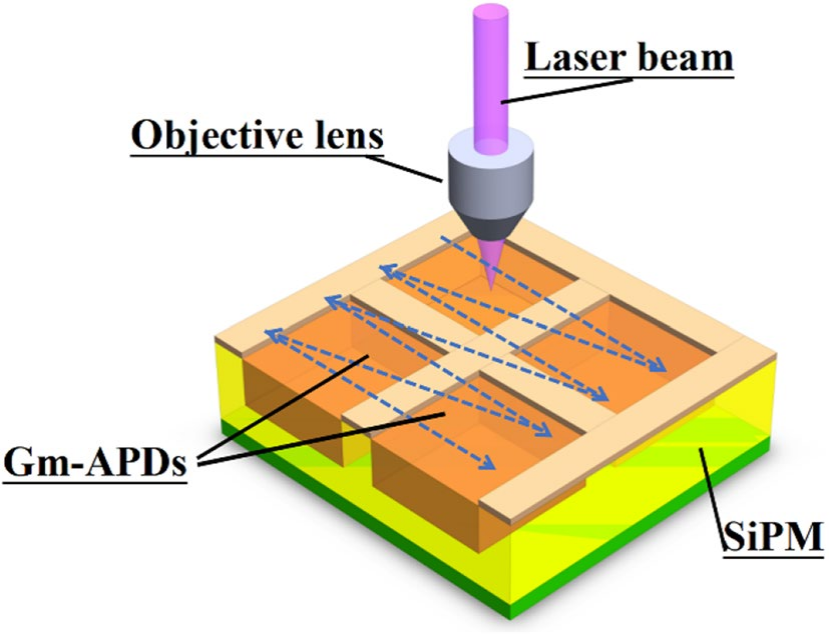
Illustration of the scanning process of the focused laser spot relative to SiPM (blue dotted lines with arrows represent the scanning route; *Gm-APD* Geiger mode avalanche photodiode).

**Figure 3:**
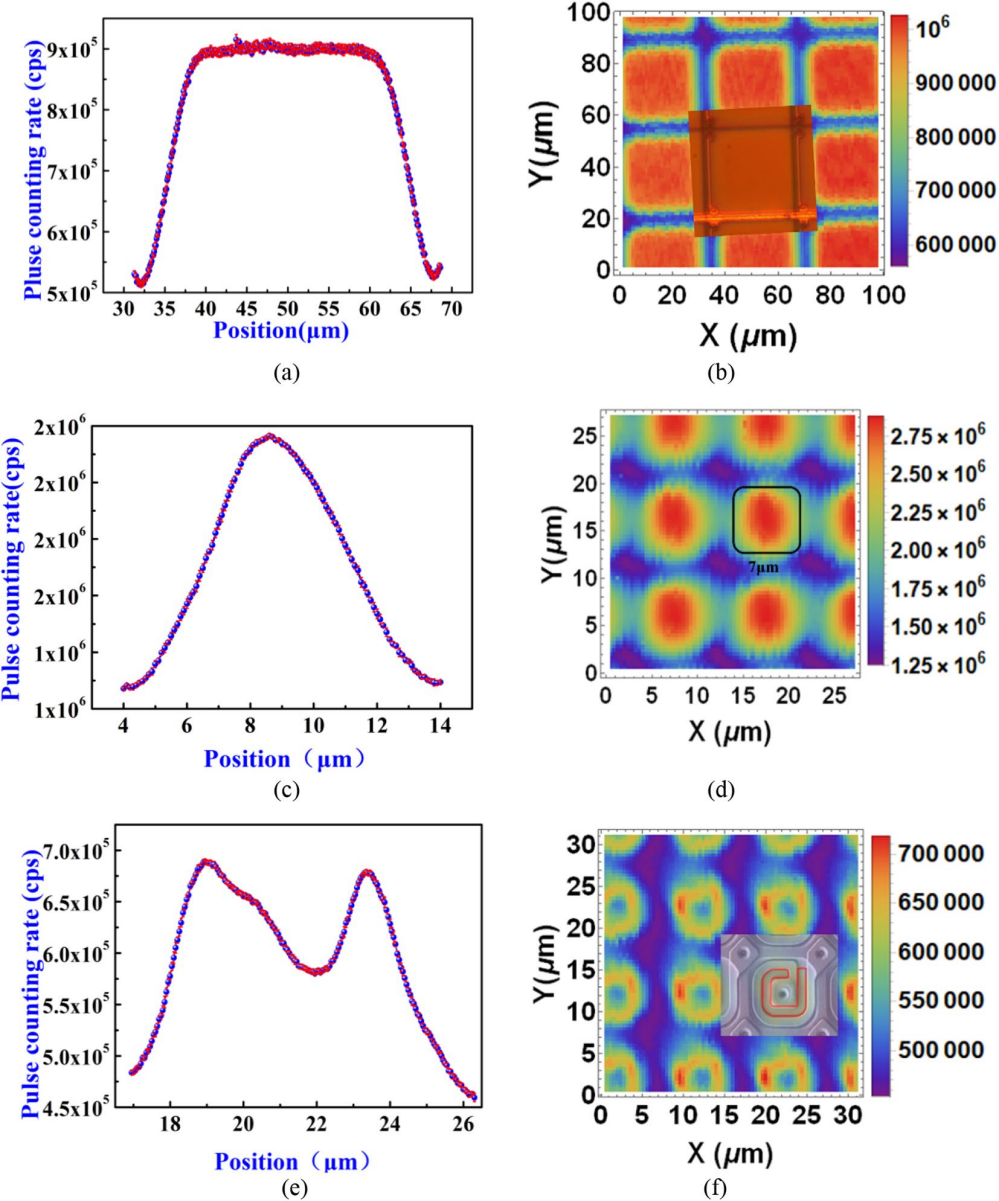
Spatial 1D and 2D distributions of the relative photoresponse (i.e.*PCR*, recorded at 0.5 p.e. threshold) of SiPMs to the single-photon-level light focal spot. (**a**) 1D and (**b**) 2D distribution of the *PCR* of the SiPM from Fondazione Bruno Kessler (FBK), Italy (model = NUV-HD-LF), with 35 μm Gm-APD cell pitch(The inset is the optical microscope photograph of the SiPM surface); (**c**) 1D and (**d**) 2D distribution of the *PCR* of the SiPM from novel device laboratory (NDL),China (model = EQR1011-1010C-T), with 10 μm Gm-APD cell pitch(the black solid square with rounded corners is the layout of the active region^29^); (**e**) 1D and (**f**) 2D distribution of the *PCR* of the Hamamatsu SiPM, Japan (model = S12571-010C) with 10 μm Gm-APD cell pitch(The inset is the secondary electron microscopy (SEM) image of the SiPM surface^18^). ((**a**,**c**,**e**) is corresponding to the function *h*(*x*, *y*) in Eq. (1)).

A diagram showing the scanning process of the focal spot relative to the photosensitive surface of the SiPM is presented in Fig. 2. As the pitch of the Gm-APD cell of the SiPMs differs, a suitable scanning step length and number of steps must be set when measuring the *PCR* of an SiPM. The spatial distribution of the *PCR* can also be called the spatial distribution of the detector’s relative photoresponse because the *PCR* directly reflects the local relative light response of the detector. The intensity of the laser pulse remained constant during the experiment, so the spatial distribution of the intensity of the focal spot reaching different SiPM surfaces should also be constant. If the size and shape of the Gm-APD cell of the SiPM as well as the spatial distribution of its photon sensitivity are known, the relative intensity spatial distribution and size of the focal spot under test, can be deduced by deconvolution method which is well known in Fourier optics or information optics^26^. Specifically, the spatial 1D and 2D distributions of the relative light intensity of the focused laser spot, can be obtained by deconvoluting the 1D and 2D *PCR* data of the SiPM and the normalized photoresponse function of the Gm-APD cell of SiPM (vide infra and Fig. 3).

As an SiPM is composed of Gm-APD cell array with a regular cell shape (mostly square) and gaps between adjacent Gm-APD cells, the photosensitive area of the Gm-APD cell in an SiPM can be described mathematically by a rectangular function or a trapezoid function. The trapezoid function considered the decreasing electric field at the edge of the photosensitive area in a Gm-APD cell^27^, by first order approximation. Hence the measured 2D relative photoresponse data, *h*(*x*, *y*), can be regarded as the convolution of a trapezoid or rectangle function *g*(*x*, *y*), and the spatial distribution function of the focal spot intensity, *f*(*x*, *y*). They are related through the expression in Eq. (1),

1 $$f(x,y) * g(x,y) + \varepsilon (x,y) = h(x,y)$$

where *x* and *y* are the coordinates in the waist cross-section of the focused laser beam perpendicular to the propagation direction of the beam, and *ε*(*x*, *y*) is the noise fluctuation of the measured data, which can be eliminated by a low-pass filtering operation. Then, according to the principle of the Fourier optics^26^, the intensity distribution function, *f*(*x*, *y*), can be obtained via deconvolution operation. Take the discrete Fourier transformation of *h*(*x*, *y*) and *g*(*x*, *y*) in Eq. (2) and (3), respectively,

2 $$H(f_{x} ,f_{y} ) = \int_{{{{ - L_{0} } \mathord{\left/ {\vphantom {{ - L_{0} } 2}} \right. \kern-\nulldelimiterspace} 2}}}^{{{{L_{0} } \mathord{\left/ {\vphantom {{L_{0} } 2}} \right. \kern-\nulldelimiterspace} 2}}} {h(x,y)e^{{i2\pi (f_{x} x + f_{y} y)}} } {\text{d}}x{\text{d}}y,$$

3 $$G(f_{x} ,f_{y} ) = \int_{{ - {{L_{0} } \mathord{\left/ {\vphantom {{L_{0} } 2}} \right. \kern-\nulldelimiterspace} 2}}}^{{{{L_{0} } \mathord{\left/ {\vphantom {{L_{0} } 2}} \right. \kern-\nulldelimiterspace} 2}}} {g\left( {x,y} \right)e^{{i2\pi (f_{x} x + f_{y} y)}} dxdy} ,$$

where *f*_x_, *f*_y_ are the variable in space frequency domain and *L*_0_ is the scanning range. Then, according to the Fourier transformation principle, we have the relationship in Eq. (4),

4 $$F(f_{x} ,f_{y} ) = {{H(f_{x} ,f_{y} )} \mathord{\left/ {\vphantom {{H(f_{x} ,f_{y} )} {G(f_{x} ,f_{y} )}}} \right. \kern-\nulldelimiterspace} {G(f_{x} ,f_{y} )}}$$

where *F*(*f*_x_, *f*_y_) is the Fourier transformation of *f*(*x*, *y*). The spatial distribution function of the relative light intensity of the focal spot, *f*(*x*, *y*), can then be obtained by the inverse Fourier transformation, as shown in Eq. (5):

5 $$f(x,y) = F^{ - 1} \left\{ {F(f_{x} ,f_{y} )} \right\}$$

The size of the focal spot can be estimated by eye in the map of *f*(*x*, *y*), while a more precise size (defined as the FWHM) of the focal spot can be obtained by function fitting of *f*(*x*, *y*). For the 1D relative light intensity distribution of a focal spot, *f*(*x*) or *f*(*y*), can be obtained by deconvolution operation of the 1D *PCR* data. And the size of the focal spot along the *x*- or *y*-direction can be obtained through unary function fitting of the deconvolution data, *f*(*x*) or *f*(*y*), respectively. As the semiconductor laser was working in the fundamental mode in this work, the output beam was a Gaussian beam, which is focused to a Bessel beam by the lens along its longitudinal axis. According to the well-known principle of optics^28^, *f*(*x*) or *f*(*y*) can be fitted by the Airy formula based on the Bessel function, as shown in Eq. (6),

6 $$I(v) = \left[ {\frac{{2J_{1} (v)}}{v}} \right]^{2} I_{0}$$

where *I*(*v*) is the intensity distribution of a focal spot, *v* is the distance relative to the extreme point of the distribution, *J*_1_(*v*) is the first-order Bessel function in the first class, and *I*_0_ is the maximum intensity of the distribution. As the measured *PCR* data includes the dark count rate of the SiPM and possible stray-photon-induced pulse count rate, an intensity shift should be added into the fit function, as shown in Eq. (7),

7 $$I(v) = \left[ {\frac{{2J_{1} (v)}}{v}} \right]^{2} I_{0} + I_{{\text{d}}}$$

where *I*_d_ is the baseline of the light intensity distribution.

## Results and discussion

From Fig. 3a,b, it is clear that the spatial distribution of the *PCR* within an Gm-APD cell (cell pitch = 35 μm) is uniform, and different Gm-APD cells can be clearly distinguished. Figure 3c,d show the 1D and 2D distribution of the *PCR* of the NDL SiPM (cell pitch = 10 μm), we can see that the Gm-APDs are still distinguishable. As the layout spacing between two neighboring Gm-APD cells is 3 μm according to Ref^29^, Fig. 3d suggests that the size of the focal spot should be smaller than 3 μm, otherwise the different Gm-APD cells would not be distinguishable. Figure 3e,f show the 1D and 2D *PCR* distribution of the Hamamatsu SiPM with 10 μm cell pitch, respectively. The details in each Gm-APD cell can still be resolved by eye (Fig. 3f). The inset in Fig. 3f shows the SEM image of the 10 μm Gm-APD cell^18^. In the inset, the region inside the red solid line is the un-shading region, contrasting to the shading region by the quenching resistor film. One can see that the outline of un-shading region (i.e. the red solid line) in the inset in Fig. 3f is basically consistent with the outline of the photosensitive region of the SPADs in the 2D map (Fig. 3f).

**Figure 4:**
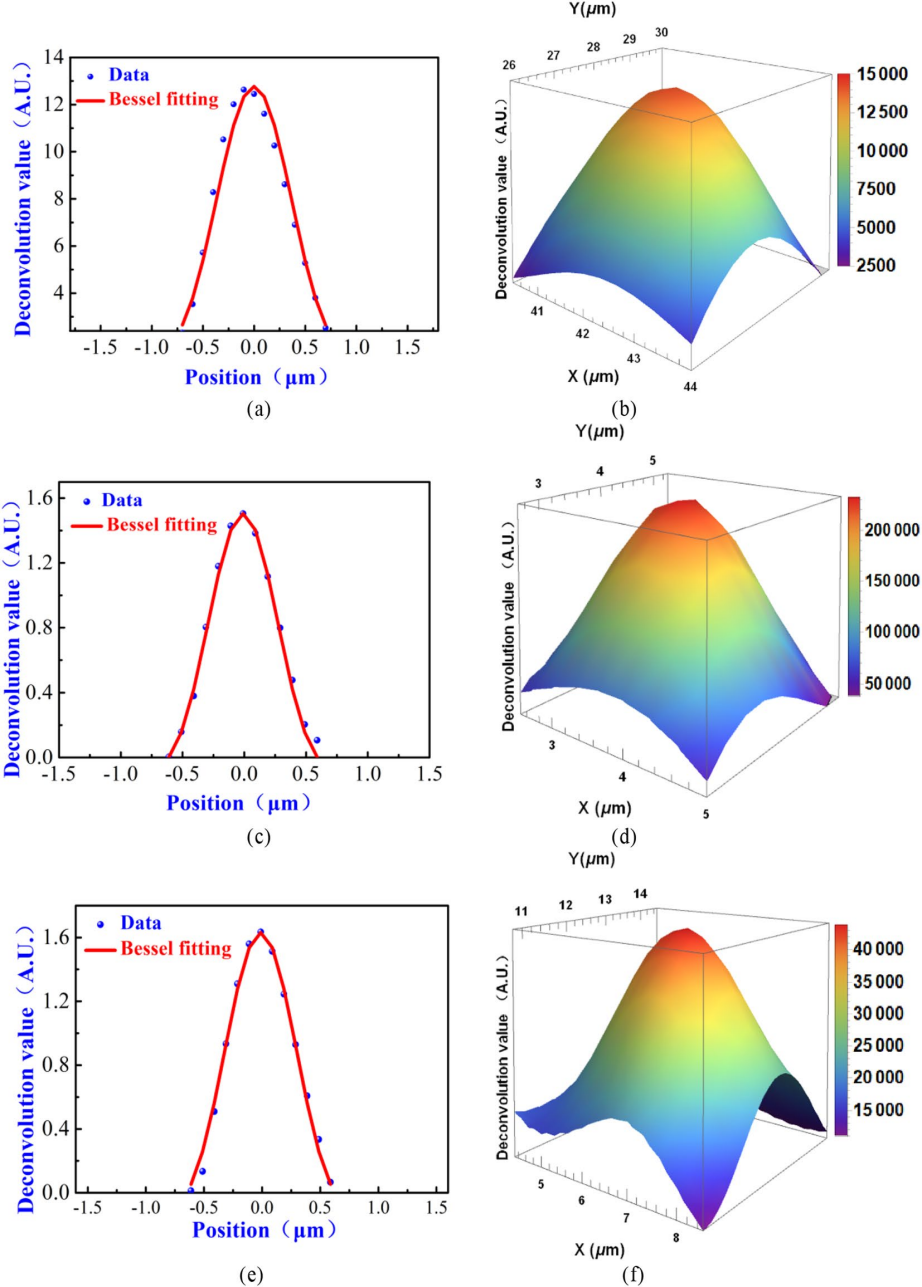
Deconvolution results of the relative photoresponse (*PCR*) of the SiPMs: (**a**) 1D and (**b**) 2D (shown in 3D-view) deconvolution results obtained by the FBK SiPM with 35 μm Gm-APD cell pitch (model = NUV-HD-LF); (**c**) 1D and (**d**) 2D (shown in 3D-view) deconvolution results obtained by the NDL SiPM with 10 μm cell pitch (model = EQR1011-1010C-T); (**e**) 1D and (**f**) 2D (shown in 3D-view) deconvolution results obtained by the Hamamatsu SiPM with 10 μm cell pitch (model = S12571-010C).

The 1D deconvolution results (Fig. 4) of the 1D spatial distributions of the *PCR* of SiPMs (Fig. 3) were obtained by Eq. (1–5) and the rectangular function model. It is clear that for each of the tested SiPM, the spatial distribution range of the focal spot intensity was on the micron scale, and the intensity distribution of the focal spot closely followed a Bessel distribution. To quantitatively evaluate the size of the focal spot, the Airy formula based on the Bessel function (see Eq. (7)) was used to fit the 1D deconvolution results. The fitting results are shown as solid red lines in Fig. 4a,c,e, and the fitting parameters are shown in Table 2. The fitting results indicated that the average FWHM of the focal spot was 0.657 μm. To more intuitively visualize the relative intensity distributions of the focal spot, the 2D deconvolution operation was performed with the data shown in Fig. 3b,d,f to obtain the 3D views of the deconvolution results (shown in Fig. 4b,d,f, respectively). The spatial distribution ranges of the focal spot under test were basically consistent with the 1D results.

**Table 2:** Fitting parameters of the focal spot under test.

Model of SiPMs	Gm-APD cell pitch (μm)	FWHM of the focal spot (μm)	Adjusted *R*^2^
FBK SiPM (NUV-HD-LF)	35	0.684 ± 0.030	0.996
NDL SiPM (EQR1011-1010C-T)	10	0.641 ± 0.034	0.997
Hamamatsu (S12571-010C)	10	0.646 ± 0.001	0.995

Table 2 shows the FWHM of the focal spot measured by the three models of SiPMs; these results are generally consistent with the nominal resolution of the objective lens of the microscope (i.e., 0.42 μm) and the focal spot size (i.e., 0.8 μm) mentioned by Anfimov et al*.* when measuring the fill factor of a SiPM via focused laser scanning^30^. The obtained FWHM result (i.e. 0.657 μm on average) are larger than the nominal focal spot resolution of the objective lens (0.42 μm), which may be due to the fact that the setup of the optical path was not adjusted to the optimal state. The adjusted *R*^2^ (i.e. the fitting determination coefficient. if the adjusted *R*^2^ is 0, the fitting effect of the model is poor; if the result is 1, the fitting is error-free.) coefficients are all close to 1, indicating that the light intensity distribution of the laser focal spot follows a Bessel distribution with high confidence.

It is worth noting that we found the different mathematical function models for describing photoresponse of Gm-APD cells in SiPM has relative little affect on the FWHM results of the focal spot (See Supplementary Information). The maximum difference of the FWHM results obtained by the two different function model (i.e. the rectangular function and trapezoid function) is 0.14 μm (See Supplementary Information). For simplicity, the rectangular function is adequate to serve as the photoresponse fucntion in our proposed method. All of the results indicate the focal spot under test is sub-micron. Therefore, the focal spot size evaluation results obtained in these experiments are reasonable.

**Figure 5:**
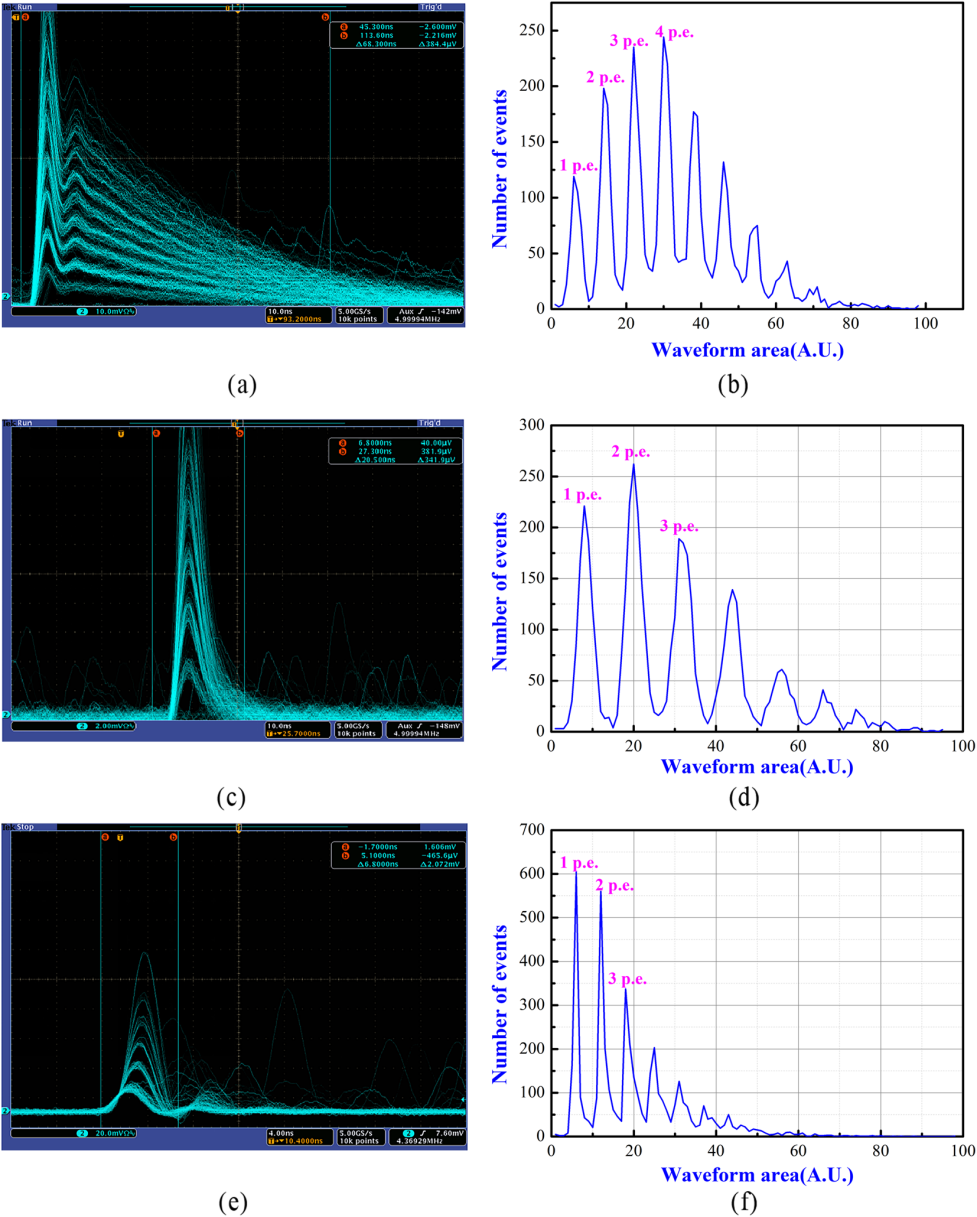
Oscillograph capture and the photon number resolution spectra of the SiPMs employed in this work. (**a**) Oscillograph showing the avalanche pulses from the FBK SiPM (model = NUV-HD-LF) under the irradiation of the focal spot; (**b**) Photon number resolution (histogram of the waveform area) spectrum of the FBK SiPM; (**c**) Waveforms of the avalanche pulses from the NDL SiPM (model = EQR1011-1010C-T) under the irradiation of the focal spot; (**d**) Photon number resolution spectrum of the NDL SiPM; (**e**) Oscillograph showing the avalanche pulses from the Hamamatsu SiPM (model = S12571-010C) under the irradiation of the focal spot; (**f**) Photon number resolution spectrum of the Hamamatsu SiPM.

To confirm that the focal spot incident on the SiPM surface is weak enough to the single-photon level, we recorded the oscilloscope capture and measured the histogram of the waveform area of the avalanche pulses output by the three kinds of SiPMs (see Fig. 5). Single-photon response pulses were clearly observed, and the pulses have a clear multiple relationship, corresponding to the number of detected photons (see Fig. 5a,c,e). Additionally, Fig. 5b,d,f shows a clear discrete photon-equivalent peak, which indicates that the most probable number of detected photons is 4, 2 and 1, respectively. The difference of detected photons is because that the photon detection efficiency (PDE) of the three SiPMs are different. Since the discrete peaks in Fig. 5b,d,f correspond to the number of detected photons, these plots are often called the photon number resolution spectra. The estimated average number of the photons hitting the surface of the SiPMs is about 8, obtained by dividing the average detected photons by the PDE of the SiPMs. Overall, the results in Fig. 5 confirm that the number of photons hitting the SiPM is at the single-photon level.

## Conclusions

This work verified that an SiPM combined with precision scanning technology can be used to indirectly measure the size of a sub-micron focal spot at the single-photon level. According to the deconvolution results from the measured 1D relative photoresponse distribution data, the average FWHM of the focal spot under test was about 0.657 μm, which is close to the resolution of the objective lens in the microscope (0.42 μm). The proposed method has two key advantages: first, it can be used to measure a sub-micron focal spot at the single-photon level, and second, the focal spot can easily be aligned with the detector. Through rapid scanning and a deconvolution operation, the spatial distribution of the intensity of a light focal spot can be obtained, which could lead to various applications in weak light focal spot detection and the development of sub-micron focal spot measurement techniques.

## Supplementary Information

Supplementary Information.
