# Author Correction: Measurement of focal light spot at single-photon level with silicon photomultipliers

**DOI:** 10.1038/s41598-022-25506-6

**Published:** 2022-12-05

**Authors:** Guoqing Zhang, Yaxian Yang, Chen Zhang, Xinyue Cao, Lina Liu, Lianbi Li, Xiaoxiang Han

**Affiliations:** 1 School of Science, Xi’an Polytechnic University, Xi’an, 710048 China; 2 School of Science, Xi’an University of Technology, Xi’an, 710048 China

Correction to: *Scientific Reports* 10.1038/s41598-022-17759-y, published online 05 September 2022

The original version of this Article contained an error in the Acknowledgments section.

“This work was supported by the National Natural Science Foundation of China (Grant Nos. 11975176), the Natural Science Basic Research Plan in Shaanxi Province of China (Grant No. 2019JM-139), the Key Research and Development Program of Shaanxi Province (No. 2020KW-011), the Fundamental Research Funds of Shaanxi Key Laboratory of Artificially-Structured Functional Materials and Devices (No. AFMD-KFJJ-21207).”

now reads:

“This work was supported by the National Natural Science Foundation of China (Grant No. 11975176), the Key Research and Development Program of Shaanxi Province (No. 2020KW-011), the Fundamental Research Funds of Shaanxi Key Laboratory of Artificially-Structured Functional Materials and Devices (No. AFMD-KFJJ-21207) and Natural Science Foundation of Shaanxi (2022JQ-660).”

The original Article has been corrected.

