# Supplementary material for: Measurement of focal light spot at single-photon level with silicon photomultipliers
Source: Sci Rep. 2022 Sep 5;12:15060. doi: 10.1038/s41598-022-17759-y (PMC9445098; doi:10.1038/s41598-022-17759-y)
Supplement: Supplementary file 1 — Supplementary Information. [file 41598_2022_17759_MOESM1_ESM.pdf]

Supplementary material

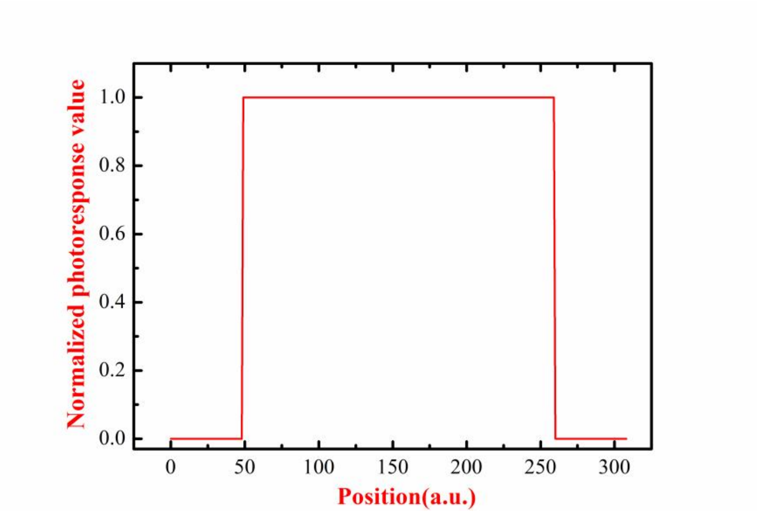

(a)

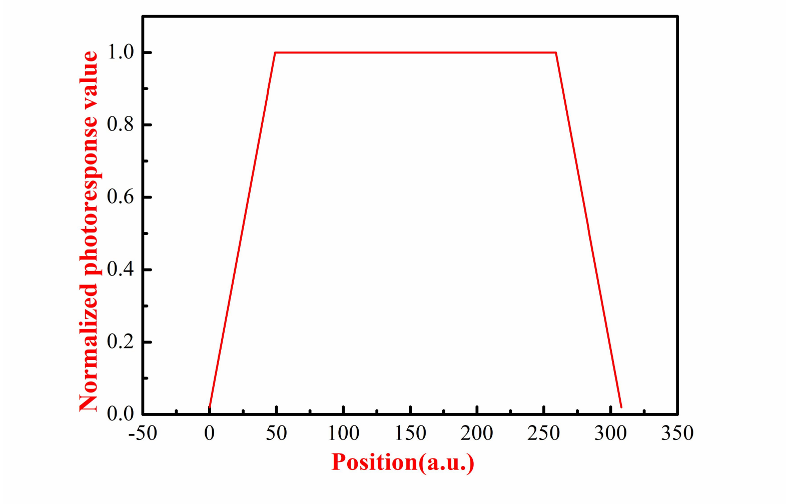

(b)

Figure 1S. The schematic diagram of the relative photoresponse function. (a)the rectangle function; (b) the trapezoid function.

Fig 1S shows the diagram of the rectangle function and the trapezoid function mentioned in the main text. The slopes of the trapezoid function were obtained according to the maximum slope of the PCR data. The trapezoid width was adjusted according to the width of the PCR data from different SiPMs.

Fig 2S shows the deconvolution results of the relative photoresponse (i.e. PCR) of the SiPMs by trapezoid function model mentioned in the main text(in the experimental setup and principle section). The results are similar to the results from the rectangle function(box-like) model.

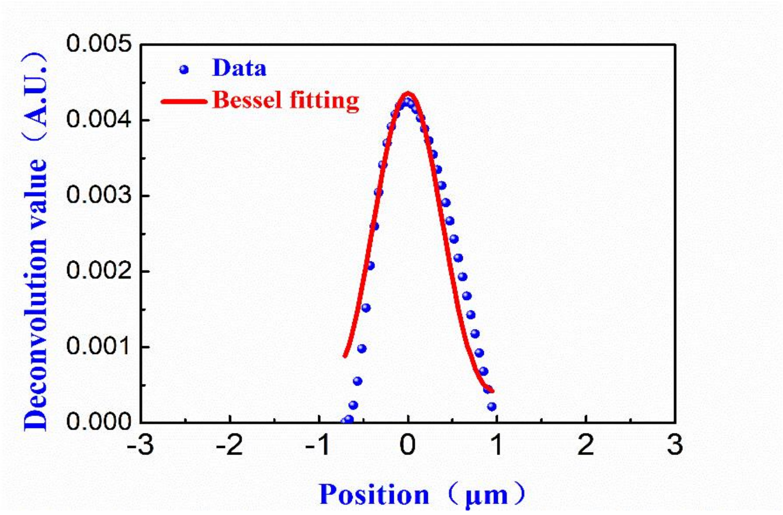

Data
Bessel fitting
Deconvolution value（A.U.）
0.005
0.004
0.003
0.002
0.001
0.000
-3
-2
-1
0
1
2
3
Position（μm）

(a)

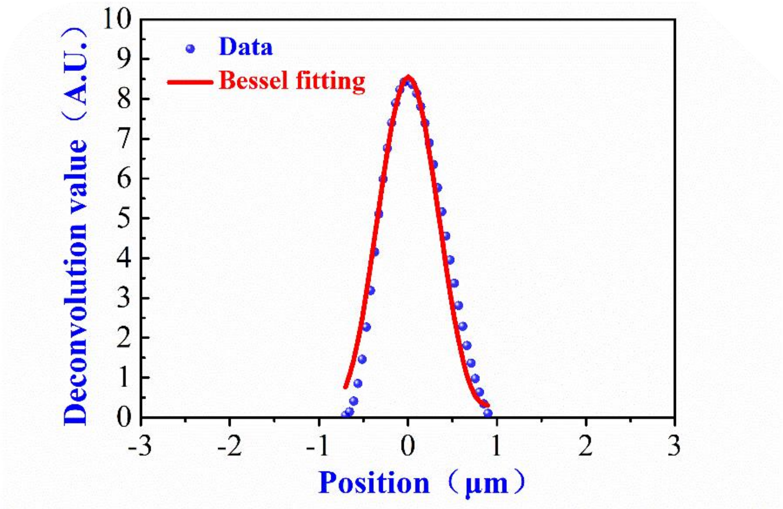

Data
Bessel fitting
Deconvolution value（A.U.）
10
9
8
7
6
5
4
3
2
1
0
-3
-2
-1
0
1
2
3
Position（μm）

(b)

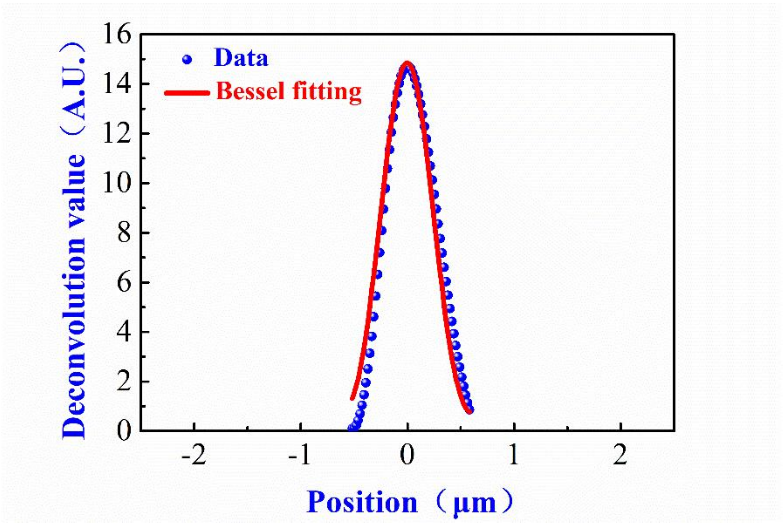

Data
Bessel fitting
Deconvolution value（A.U.）
16
14
12
10
8
6
4
2
0
-2
-1
0
1
2
Position（μm）

(c)

Figure 2S. Deconvolution results of the relative photoresponse (i.e. PCR) of the SiPMs by trapezoid function model. (a) 1D deconvolution values obtained by the FBK SiPM with 35 μm GM-APD cell pitch (model =NUV- LF-HD); (b) 1D and deconvolution values obtained by the NDL SiPM with 10 μm GM-APD cell pitch (model = EQR1011-1010C-T); (c) 1D and deconvolution values obtained by the Hamamatsu SiPM with 10 μm GM-APD cell pitch (model = S12571-010C)

SiPM Model	FWHM of the focal spot under test (μm)	Adjusted R^2
FBK SiPM (NUV-LF-HD)	0.781±0.009	0.970
NDL SiPM (EQR1011-1010C-T)	0.748±0.001	0.987
Hamamatsu(S12571-010C)	0.532±0.003	0.992

Table 1S. Fit results of the deconvolution data obtained from the trapezoid function model.

From Table 1S we can see that the FWHM of the focal spot changes by different SiPMs. However, the maximum deviation of the FWHM results obtained by the two different function model(i.e. the rectangular function and the trapezoid function) is 0.14 μm.
